# Primary antibiotic resistance and associated mechanisms in *Helicobacter pylori* isolates from Senegalese patients

**DOI:** 10.1186/1476-0711-12-3

**Published:** 2013-01-08

**Authors:** Abdoulaye Seck, Christophe Burucoa, Daouda Dia, Mouhamadou Mbengue, Manuella Onambele, Josette Raymond, Sebastien Breurec

**Affiliations:** 1 Institut Pasteur, Unité de Biologie Médicale et Environnementale, 36 avenue pasteur, Dakar, BP220, Senegal; 2 EA 4331 LITEC, Université de Poitiers, CHU de Poitiers, Laboratoire de Bactériologie-Hygiène, 2, rue de la Miletrie, Poitiers, BP 577, 86021, France; 3 Département de Gastro-entérologie, Centre Hospitalier Le Dantec, 30 avenue Pasteur, BP3001, Dakar, Senegal; 4 Unité Postulante de Pathogénèse de Helicobacter, Institut Pasteur, 25-28 rue du Docteur Roux, 75724, Paris Cedex 15, France

**Keywords:** *Helicobacter pylori*, Levofloxacin, Clarithromycin, Antibiotic resistance, Senegal

## Abstract

**Background:**

Antibiotic combination therapy for *Helicobacter pylori* eradication must be adapted to local resistance patterns, but the epidemiology of *H. pylori* resistance to antibiotics is poorly documented in Africa. The aim was to determine the antibiotic resistance rates, as well as the associated molecular mechanisms, of strains isolated in Dakar, Senegal.

**Methods:**

One hundred and eight *H. pylori* strains were isolated between 2007 and 2009 from 108 patients presenting with upper abdominal pain to the Gastroenterology Department of Le Dantec Hospital. Antimicrobial susceptibility testing was performed for amoxicillin, clarithromycin, metronidazole, levofloxacin and tetracyclin using the E-test method. Mutations in the 23S rRNA gene of clarithromycin-resistant strains and in *gyrA* and *gyrB* of levofloxacin-resistant strains were investigated.

**Results:**

Isolates were characterized by no resistance to amoxicillin (0%), tetracycline (0%), and very low rate of resistance to clarithromycin (1%), but a high rate of resistance to metronidazole (85%). The clarithromycin-resistant strain displayed the A2143G mutation. A worrying rate of levofloxacin resistance was detected (15%). N87I and D91N were the most common mutations in the quinolone-resistance-determining region of *gyrA*.

**Conclusions:**

The first-line empirical regimen for *H. pylori* eradication in Senegal should include clarithromycin. Increasing rates of fluoroquinolone resistance detected should discourage the use of levofloxacin-containing regimens without prior antimicrobial susceptibility testing.

## Background

*Helicobacter pylori* is associated with severe gastroduodenal disorders, including peptic ulcer disease, gastric adenocarcinoma, and gastric mucosa-associated lymphoid tissue lymphoma [1]. About 15% of infected people will develop a peptic ulcer and 1-3% a gastric malignancy during their lifetime. All guidelines worldwide recommend the eradication of *H. pylori* in symptomatic patients [2]. This treatment commonly consists of triple-agent therapy with a double-dose proton pump inhibitor (PPI) and two antibiotics chosen from amoxicillin, clarithromycin and metronidazole, for 7 to 14 days, yielding an eradication rate ranging from 70% to 80%. Antibiotic resistance is the main cause of treatment failure. In particular, clarithromycin resistance can lead to a 70% decrease in efficacy [3]. Bismuth-containing four-drug regimens or, if the latter are unavailable, sequential treatment or a non-bismuth four-drug regimen, is recommended for first-line empirical treatment in areas of high clarithromycin resistance. Levofloxacin-containing triple therapy should be preferred when these treatments fail [2]. Clarithromycin resistance is mainly due to point mutations in the 23S ribosomal RNA (rRNA) gene. A2143G and A2142G are the most frequent mutations [4]. Resistance to quinolones is mainly due to mutations in the quinolone-resistance-determining region (QRDR) of the *gyrA* gene, coding for the A subunit of the DNA gyrase, at codons 86, 87, 88 and 91 [3]. Very few data are available on *H. pylori* resistance to antibiotics in Africa [5-9]. Here we examined the prevalence of *H. pylori* resistance to antibiotics, and the associated molecular mechanisms, in symptomatic African patients in Dakar (Senegal).

## Methods

### Study population

A total of 108 *H. pylori* strains were isolated between 2007 and 2009 from 108 patients presenting with upper abdominal pain to the Gastroenterology Department of Le Dantec Hospital (Dakar, Senegal). Median age was 42.0 years (mean 45.3 years; interquartile range (IQR) 31.0–56.6 years; range, 18–93 years), and 55% of the patients (n=59) were males. The predominant ethnic groups were Wolof (37%), Fulani (16%), and Serer (14%), in keeping with the ethnic distribution in Senegal. On the basis of endoscopic findings, 30 patients had gastritis only, 63 had ulcerated lesions, and 15 had suspicion of neoplasia. All cases of suspected neoplasia were histologically confirmed as gastric cancer.

No patient had previously received anti-*H. pylori* therapy. Three biopsy samples were taken from the antrum and three from the fundus during upper gastrointestinal endoscopy. One biopsy sample from each site was cultured for *H. pylori* isolation, and the other four were fixed and processed for histological analysis. All endoscopes and accessory devices were decontaminated after every endoscopy, according to local written procedures. On the basis of endoscopic findings, patients were classified as having gastritis only, ulcerated lesion, or suspicion of neoplasia. Written informed consent was obtained from all the patients and the study protocol was approved by the Senegalese national ethics committee.

### Histology

Histopathological diagnoses were performed according to the updated Sydney System [10], and to the Vienna classification for dysplasia [11].

### *H. Pylori* culture and antimicrobial susceptibility testing

*H. pylori* culture was performed using Columbia agar plates with 10% (v/v) defibrinated horse blood and an *H. pylori* selective antibiotic supplement (Oxoid, Basingstoke, UK) containing vancomycin (10 mg/L), cefsulodin (5 mg/L), trimethoprim (5 mg/L) and amphotericin B (5 mg/L). The plates were incubated for up to 10 days at 37°C in microaerophilic conditions (GENbag, Biomerieux). *H. pylori* was identified by colony and microscopic morphology, and by positive urease, catalase and oxidase tests. Antibiotic susceptibility was determined from a mixture of colonies from antrum and fundus samples with the E-test method (Biomérieux, Marcy l’Etoile, France) using Mueller–Hinton agar supplemented with 10% horse blood as previously described [12]. According to CLSI breakpoints [13], the resistance breakpoints were 0.5 mg/L for amoxicillin (EUCAST [14], ≥ 0.12 mg/L), 1 mg/L for clarithromycin (EUCAST, ≥ 0.5 mg/L), 2 mg/L for tetracycline (EUCAST, ≥ 1 mg/L), 1 mg/L for levofloxacin (similar to the EUCAST value), and 8 mg/L for metronidazole (similar to the EUCAST value).

### DNA extraction and mutation analysis of the 23S rRNA, *gyrA* and *gyrB* genes

Genomic DNA was extracted with the QIAmp kit (Qiagen, Courtaboeuf, France). The QRDR region of *gyrA* was sequenced using primers gyrA5 and gyrA2 in levofloxacin-resistant strains [4]. If no mutation was detected, the full-length *gyrA* and *gyrB* genes were sequenced as previously described [4] and were compared with the sequences from five randomly selected levofloxacin-sensitive isolates. Point mutations in the 23S rRNA gene were investigated by using Scorpion PCR in strains resistant to clarithromycin [12].

### Statistical analysis

The chi-square test for trend was used to compare rates of resistance to antibiotics during the study period. P values of ≤0.05 were considered statistically significant. All analyses were performed by using STATA 12.0 (Stata Corporation, College Station, Tex.).

## Results

### Patients and antibiotic susceptibility testing

One hundred eight *H. pylori* isolates were cultured from gastric biopsy samples obtained from volunteers during gastroduodenal endoscopy at Le Dantec Hospital (Dakar, Senegal) between 2007 and 2009.

According to EUCAST and CLSI breakpoints, all isolates tested were susceptible to amoxicillin (MIC range, <0.016-0.047 mg/L), tetracycline (MIC range, <0.016-0.75 mg/L) and clarithromycin (MIC range, <0.016-0.47 mg/L), except for one clarithromycin-resistant strain (MIC>256 mg/l). The isolates were characterized by high rates of resistance to metronidazole (MIC range, <0.016->256 mg/L) (85%, n=92) and intermediate rates of susceptibility to levofloxacin ((MIC range, <0.002->32 mg/L) (85%, n=92) (Table 1). The clarithromycin-resistant strain was resistant to both levofloxacin (MIC>32 mg/L) and metronidazole (MIC>256 mg/L). Twelve of the 16 levofloxacin-resistant strains were also resistant to metronidazole. No significant difference in the prevalence of resistance to antibiotics was observed during the study period (P>0.5).

**Table 1:** **Resistance to antibiotics of *H. pylori *isolates in Dakar, Senegal, from 2007 to 2009**

	**n (%)**	**MIC (mg/L)**	
		**Median**	**Range**
Amoxicillin	0	<0.016	<0.016-0.047
Tetracycline	0	<0.016	<0.016-0.75
Levofloxacin	16 (15)	0,125	<0.002->32
Metronidazole	92 (85)	>256	<0.016->256
Clarithromycin	1 (1)	<0.016	<0.016->256

### Mutation of the 23S rRNA gene in the clarithromycin-resistant strain and of *gyrA* and *gyrB* in levofloxacin-resistant strains

Only one strain was resistant to clarithromycin. Resistance was due to a point mutation at nucleotide position 2143 (A2143G) of the 23S rRNA gene. Among the 16 levofloxacin-resistant strains, 13 harbored a specific mutation in the QRDR of the *gyrA* gene, previously reported to confer resistance to fluoroquinolones. The amino acid positions concerned were 87 and 91: N87I, n=7 (MICs>32 mg/l); D91N, n=4 (MIC range, 4–16 mg/l); D91G, n=1 (MIC=2 mg/l); D91Y, n=1 (MIC>32 mg/l). As three strains with MICs of 8 mg/l displayed no mutations in the QRDR of *gyrA*, the entire *gyrA* and *gyrB* genes of these strains were sequenced. No mutation was detected.

## Discussion

Antibiotic resistance is the most important factor responsible for the declining success rate of *H. pylori* eradication therapy. Surveillance of *H. pylori* antibiotic resistance is mandatory in order to adapt the antibiotic combination to local resistance patterns.

The rates of *H. pylori* resistance to amoxicillin (0%) and metronidazole (85%) observed in Senegal were in keeping with those reported in this country in 1999–2000 (0% and 90%, respectively) [8]. In contrast, resistance to levofloxacin rose from 0% in 1999–2000 to 15% in 2007–2008, mirroring the situation in several European and Asian countries, with increases from 3% in 1999 to 15% in 2004 in France, from 11% in 2003 to 22% in 2005 in Germany, from 3% in 1998 to 12% in 2003 in Taiwan, and from 0% in 1987 to 33% in 2003 in South Korea [15-19]. This probably reflects the increasing use of quinolones in these countries [20,21], highlighting the importance of appropriate use of this class of antibiotics to limit the development of antimicrobial resistance. Interestingly, a significant increase in ciprofloxacin resistance has also been observed in *Escherichia coli* isolates from Senegalese patients with community-acquired urinary tract infections (10% in 2004, 22% in 2006) [22].

The lack of resistance to amoxicillin and tetracycline in this study indicates that *H. pylori* resistance to these agents is probably exceptional (0 to 1.3%) and low (0 to 4.4%), respectively, whatever the continent [21,23]. The high prevalence of resistance to these antibiotics observed in some developing and emerging countries probably reflects inadequate laboratory facilities [5-7,9] (e.g. 6% in Ethiopia, 6.6% in Bangladesh, 39.0% in Brazil, 59% in Iran for resistance to amoxicillin; 9.0% in Brazil, 15.0% in Bangladesh, 27.0% in Chile for resistance to tetracyclin). In our study, only one strain was resistant to clarithromycin (MIC>256 mg/l), likely reflecting low macrolide consumption, although no data on antibiotic consumption for clarithromycin but also erythromycin and azithromycin are available for Senegal. This resistance was due to a point mutation at nucleotide position 2143 (A2143G) of the 23S rRNA gene, a well-known mutation described worldwide. Resistance to clarithromycin is increasing in most countries in Central, Western and Southern Europe, as well as in East Asia, and has now reached more than 20% in these areas. Clarithromycin-containing regimens should thus be recommended for first-line empirical *H. pylori* eradication therapy.

The prevalence of resistance to levofloxacin was 15% in Senegal. In the 16 levofloxacin-resistant strains, N87I and D91N were the most common mutations in the QRDR region of *gyrA*. The major mutation (N87I) has been detected sporadically worldwide [16], indicating that local founder effects have resulted in local propagation of resistant clones. D91N is a well-known mutation detected worldwide [4]. As three strains with levofloxacin MICs of 8 mg/l displayed no mutations in the QRDR of *gyrA*, we sequenced the entire *gyrA* and *gyrB* genes of these strains but found no mutations. This points to the presence of another mechanism such as reduced drug accumulation [4]. The Maastricht IV/Florence Consensus Report recommends that empirical use of levofloxacin should be abandoned when the prevalence of resistance reaches 15–20% [2]. Levofloxacin-containing therapy should therefore only be used in Senegal if compatible with the antimicrobial resistance pattern of the strain.

The worldwide prevalence of resistance to metronidazole ranges from 20–40% in Europe and the USA, to 50–80% in developing countries, Iran, India and Egypt displaying the highest rates of resistance (80 to 100%) [6,21,24,25]. Metronidazole is used extensively to treat parasitic diseases in tropical countries, which probably explains the higher prevalence of resistance to this drug. Although standard metronidazole susceptibility testing lacks reproducibility, trends of low, medium, or high prevalence rates observed at a population level seem real. Then, metronidazole-containing triple therapy should not be recommended for first-line empirical *H. pylori* eradication therapy. It should be used within bismuth-based quadruple regimen [26] or sequential treatment for 14 days [27].

## Conclusions

Even though our findings may not be representative of the overall situation, it should be noted that the study took place in one of the main tertiary hospitals in Senegal, with a large geographical origin of the patients attending the study health facility. The data reported here are particularly important, given the difficulty of carrying out such studies in countries with inadequate healthcare systems and the need to choose antibiotic combinations for *H. pylori* eradication according to local resistance patterns. Clarithromycin-containing regimens should be recommended for first-line empirical *H. pylori* eradication therapy. Increasing rates of fluoroquinolone resistance detected in Senegal should discourage the use of levofloxacin-containing regimens without prior antimicrobial susceptibility testing. N87I and D91N were the most common mutations in the *H. pylori gyrA* QRDR.

## Abbreviations

PPI: Proton pump inhibitor; rRNA: 23S ribosomal RNA; QRDR: Quinolone-resistance-determining region.

## Competing interests

The authors have no competing interests to declare.

## Authors’ contributions

Conceived and designed the experiments: AS, CB, MM, JR; SB. Performed the experiments: AS, CB, MO, SB. Analyzed the data: AS, CB, MO, JR, SB. Contributed reagents/materials/analysis tools: AS, CB, DD, MM, SB. Wrote the paper: AS, SB. Found funding: SB, JR. All authors read and approved the final manuscript.
